# Tipping the balance in histone supply puts genome stability at stake

**DOI:** 10.1038/s44318-024-00112-6

**Published:** 2024-05-02

**Authors:** Charlène Renaud-Pageot, Geneviève Almouzni

**Affiliations:** Institut Curie, CNRS, PSL Research University, Sorbonne University, Nuclear Dynamics Unit, Équipe Labellisée Ligue contre le Cancer, Paris, France

**Keywords:** Cell Cycle, Chromatin, Transcription & Genomics

## Abstract

New work identifying an H3-H4 chaperone as factor restricting localization of CENP-A underscores the importance of balanced histone levels for maintaining proper chromosome segregation.

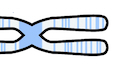

## Teaser

Specific localization of the histone H3 variant CENP-A at centromeres is key for faithful chromosome segregation during cell division, with CENP-A mislocalization being associated with chromosomal instability and cancer. New work in *The EMBO Journal* identifies the H3-H4 chaperone DNAJC9 as a factor restricting CENP-A localization, underscoring the importance of balancing H3 and CENP-A histone levels for ensuring proper CENP-A localization and maintaining genome stability.

Histones exist as distinct variants marking chromatin with unique properties. Dedicated histone chaperones regulate the distribution of each variant within the genome. As a prime example of this regulation, the presence at centromeres of a specific variant of histone H3, termed CENP-A (Centromere Protein A) in mammals, is central to direct kinetochore assembly during cell division. The specific deposition of CENP-A into centromeric chromatin involves a dedicated histone chaperone called HJURP (Dunleavy et al, 2009; Foltz et al, 2009). However, ectopic incorporation of CENP-A at non-centromeric regions occurs upon an imbalance in the proportions of histone H3 variants available, as shown in human cells overexpressing CENP-A (Lacoste et al, 2014; Athwal et al, 2015; Nye et al, 2018). Overexpression of CENP-A also leads to mitotic defects and chromosomal instability (CIN), possibly due to the trapping of kinetochore components away from the native centromeres by ectopic CENP-A nucleosomes (Shrestha et al, 2017). CENP-A overexpression and CIN are common characteristics of aggressive cancers that can impact tumor evolution and response to therapy (Renaud-Pageot et al, 2022). A critical question was whether factors that can perturb the specific localization of CENP-A without acting on CENP-A levels could also lead to a similar outcome, an important aspect to consider more broadly in cancer.

Previous studies found that when CENP-A was overexpressed, its mislocalization depended on DAXX (Lacoste et al, 2014) or HIRA (Nye et al, 2018), histone chaperones that are normally involved in the deposition of the H3.3 histone variant. Consistent with the view that overexpressed CENP-A may have saturated its chaperone HJURP, re-establishing the balance between levels of CENP-A and HJURP was able to abrogate ectopic incorporation of CENP-A (Nye et al, 2018). Thus, the dosage of CENP-A relative to its dedicated chaperone proved important to restrict its localization at the centromere. CENP-A mislocalization also occurred in human cells upon depletion of CHAF1B (p60), a subunit of the CAF-1 complex involved in the deposition of the histone variants H3.1 and H3.2. Here, the global defect in H3.1/H3.2 deposition upon CHAF1B depletion may have allowed the mislocalization of CENP-A, again through the H3.3 chaperone DAXX (Shrestha et al, 2023).

Two major questions ensue: first, can a reduction of the proportions of non-centromeric H3 variants cause CENP-A mislocalization, and second, what factors are involved in such deregulation? In a recent *EMBO Journal* article, Balachandra et al (2024) investigated these questions, and identified key factors that either promote or prevent CENP-A mislocalization in human cells. They performed a genome-wide RNAi screen, using image-based detection of CENP-A enrichment in chromatin as readout for mislocalized CENP-A. Amongst their top candidates, they found factors regulating deposition of other H3 variants, in line with an important role of H3-H4 homeostasis in preventing CENP-A mislocalization. The authors identified CHAF1A/B as one of the top candidates, consistent with previous findings (Shrestha et al, 2023). Most importantly, their screening revealed a new potential regulator, DNAJC9 (member of the J-domain containing heat shock protein HSP40 family), a co-chaperone that assists proper folding of non-centromeric histone H3 variants (Hammond et al, 2021). Balachandra and colleagues confirmed DNAJC9’s importance by depleting it, or by mutating its catalytic J-domain required for proper H3-H4 folding. Indeed, DNAJC9 depletion or loss-of-function both led to stable incorporation of CENP-A particles at non-centromeric chromatin in HeLa as well as RPE-1 cell lines. Notably, the authors did not observe higher CENP-A mRNA levels, indicating that CENP-A deregulation does not occur at the transcriptional level. Furthermore, CENP-A mislocalization phenotypes were comparable in cells depleted for DNAJC9 or overexpressing CENP-A: both displayed weakened kinetochores at native centromeres and increased chromosomal instability with higher incidence of micronuclei and defective chromosome segregation.

Finally, Balachandra et al (2024) also explored the interactome of CENP-A in DNAJC9-depleted cells. They found increased CENP-A association with MCM2, a DNA replication-associated H3 chaperone. MCM2 likely contributes to CENP-A mislocalization since co-depletion of both MCM2 and DNAJC9 abrogated CENP-A mislocalization. Since MCM2 has been implicated in the recycling of all H3 (Groth et al, 2007), it would be interesting to determine if its effect on CENP-A misincorporation also involves recycling or new deposition of ectopic CENP-A.

**Figure 1:**
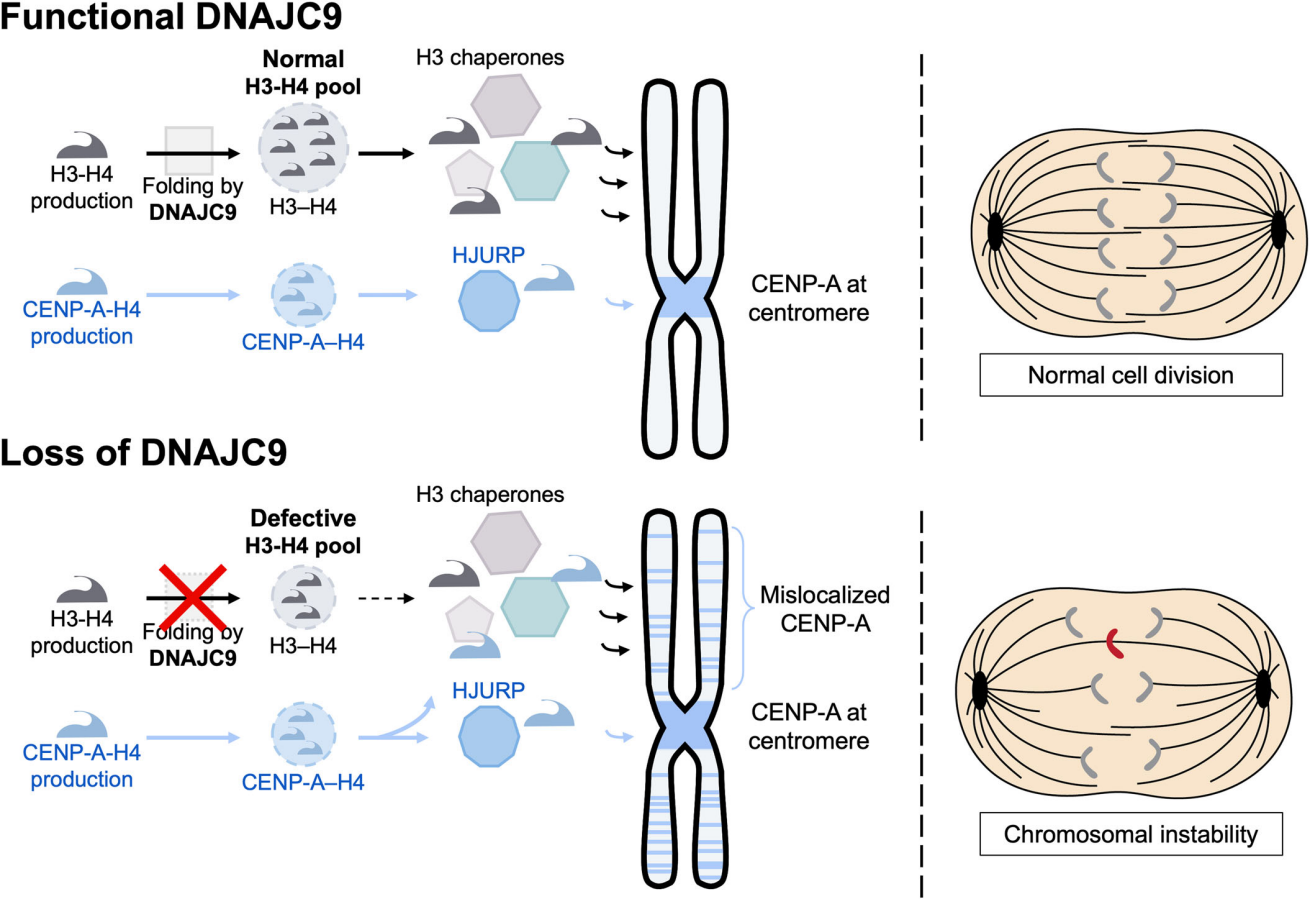
Model for DNAJC9 importance in H3-H4 supply with impact on CENP-A localization and chromosome segregation. In the presence of functional DNAJC9, a proper balance of folded H3-H4 and CENP-A-H4 pools allows CENP-A to localize at centromere, ensuring proper centromere function and chromosome segregation (top panel). Loss of DNAJC9 reduces folded H3-H4 dimers, tipping the balance in histone supply towards aberrant deposition of CENP-A, and chromosomal instability (bottom panel).

Collectively, the study by Balachandra et al (2024) demonstrates that disrupting the function of DNAJC9, which normally supplies folded H3.1-H4, H3.2-H4, and H3.3-H4 dimers, can cause CENP-A mislocalization. A general model for DNAJC9’s importance in H3-H4 supply with impact on CENP-A localization and chromosome segregation is depicted in Fig. 1. Upon reduction of the pool of folded H3-H4 dimers, there would be a higher probability of inappropriate interactions between H3-H4 chaperones and CENP-A. In turn, the chaperones would deposit CENP-A at ectopic chromosomal sites. This model is further supported by the fact that partial reduction of H3.3 levels in HeLa cells also led to CENP-A mislocalization. Balachandra et al thus unveil not only the importance of a novel factor, DNAJC9, for restricting CENP-A at centromeres, but also open new avenues for how to consider the network of chaperones and H3 variants in cancer. Indeed, CENP-A mislocalization may be a broader issue, if it depends on imbalance in the proportions of soluble variants. This imbalance can have multiple origins comprising not only overexpression of CENP-A, but also interference in the H3-H4 histone supply chain. Forthcoming research considering the histone chaperone/variant balance in patient tumors may provide avenues to better identify vulnerabilities with a therapeutic potential.
